# Treatment with CNX-011-67, a novel GPR40 agonist, delays onset and progression of diabetes and improves beta cell preservation and function in male ZDF rats

**DOI:** 10.1186/2050-6511-14-28

**Published:** 2013-05-21

**Authors:** Nagesh Gowda, Anilkumar Dandu, Jaideep Singh, Sanghamitra Biswas, Vijaya Raghav, Mudigere N Lakshmi, Pavagada C Shilpa, Venkategowda Sunil, Ashokkumar Reddy, Manojkumar Sadasivuni, Kumaraswamy Aparna, Mahesh Kumar Verma, Yoganand Moolemath, Mammen O Anup, Marikunte V Venkataranganna, Baggavalli P Somesh, Madanahalli R Jagannath

**Affiliations:** 1 Connexios Life Sciences Pvt Ltd, #49, “SHILPA VIDYA” 1st Main, 3rd Phase, JP Nagar, Bangalore 560078, India

**Keywords:** GPR40, Zucker diabetic fatty rats, PDX1, GSIS, Type 2 diabetes, Rat islets, Glucolipotoxicity, Insulin content

## Abstract

**Background:**

The role of G protein-coupled receptor (GPR40), which is highly expressed in pancreatic beta cells, has been studied extensively in the amelioration of beta cell dysfunction in T2D using rat and mouse islets, beta cell lines and in animal models of diabetes. But its potential as a therapeutic target has not been fully explored. This aim of the study is to evaluate the therapeutic potential of CNX-011-67, a highly selective, potent and orally bioavailable GPR40 agonist, in controlling diabetes and other metabolic parameters.

**Methods:**

Seven week old male ZDF rats were treated with either vehicle or CNX-011-67, 5 mg/kg twice daily, for seven weeks. The animals were subjected to oral glucose tolerance and insulin tolerance tests. Plasma glucose, insulin, triglyceride, HbA1c, fructosamine and free fatty acids were measured at selected time points. Pancreas from control and treated animals were subjected to insulin and pancreatic and duodenal homeobox 1 (PDX1) immunohistochemistry and were also evaluated by electron microscopy. Also the potential impact of CNX-011-67 on islet insulin secretion, content, ATP levels and markers of both glucose oxidation, beta cell health in rat islets under chronic glucolipotoxic conditions was evaluated.

**Results:**

Treatment of male ZDF rats with CNX-011-67 for 7 weeks significantly enhanced insulin secretion in response to oral glucose load, delayed the onset of fasting hyperglycemia by 3 weeks, reduced nonfasting glucose excursions, fasting free fatty acids and triglyceride levels. A significant increase in PDX1 expression and insulin content and reduction in plasma fructosamine, HOMA-IR, and beta cell apoptosis were observed. CNX-011-67 improves glucose mediated insulin secretion, insulin gene transcription and islet insulin content in cultured rat islets under chronic glucolipotoxic condition. Also enhanced glucose oxidation in the form of increased islet ATP content and overall improvement in beta cell health in the form of reduced expression of stress markers (TXNIP and CHOP mRNA) were observed.

**Conclusions:**

These findings, suggest that long-term oral therapy with CNX-011-67 could be of clinical value to provide good glycemic control and improve islet beta cell function.

## Background

Progressive insulin resistance and loss of beta cell function and mass are primary characteristics of type 2 diabetes mellitus. There is thus a need for a robust anti-diabetic therapy that not only provides good glycemic control but also enhances beta cell preservation and thus delays progression of the disease. Several therapeutic approaches have been adopted to prevent the onset and progression of defects in beta-cell function [1-3].

GPR40, Free Fatty Acid Receptor 1, highly expressed in human pancreatic beta cells, brain and endocrine cells of the gastrointestinal tract is activated by medium and long chain saturated and unsaturated fatty acids [4,5]. GPR40 is coupled to Gαq/11 and specific ligands like oleic acid induce an increase in cytosolic [Ca^2+^] and consequent insulin secretion at stimulatory glucose concentration in a Phospholipase C (PLC) and L-type Ca^2+^ channel dependent manner [6]. Several small-molecule agonists of GPR40 have been reported to enhance glucose-dependent insulin secretion and reduce blood glucose in mice and rodents [7-10] and also in humans [11,12].

Male ZDF rats become hyperlipidemic, hyperinsulinemic and insulin resistant by 7–8 weeks of age and subsequently hyperglycemia sets in the presence of marked hyperinsulinemia. As these animals are initially euglycemic they can be employed to investigate the delay in onset and subsequent control of progression of diabetes by therapeutic agents [13,14].

CNX-011-67 is a highly potent and selective small molecule agonist with an EC50 of 0.24 nM towards human GPR40 (Additional file 1: Figure S1). The objective of the present study was to assess the therapeutic potential of CNX-011-67 on onset and control of hyperglycemia, as well as on beta cell preservation in male ZDF rats.

## Methods

### Reagents

Glucose was estimated using Accu-check glucometer (Roche Diagnostics, Germany), HbA1c using DCA Vantage™ Analyzer (Siemens Healthcare, USA) and fructosamine using a FAR kit (FAR Diagnostics, Italy). Ultra-sensitive insulin ELISA kit (Crystal Chem Inc, USA) was used to determine plasma insulin levels. Circulating serum TG and plasma FFA were estimated using TAG estimation kit (Diasys Diagnostics system, Germany) and Randox kit (Randox Laboratories, UK) respectively. Triton-X and Fluromount were procured from Sigma-Aldrich.

### Animals

Six week old male ZDF rats (Charles River Indianapolis, IN) were housed 4 per cage under a 12-h light/dark cycle with free access to feed (rodent diet DI2450B, Research diets, US) and water, acclimatized for one week, randomized into vehicle and treatment groups and then administered vehicle (water) or CNX-011-67 (5 mg/kg, b.wt, dissolved in water, p.o. twice daily), respectively, for seven weeks. The dose was selected based on a PK/PD study in Wistar rats in which ED50 was achieved at 5 mg/kg b.wt. Body weight, and fed and fasting blood glucose were measured at weekly intervals while feed intake was monitored daily and reported weekly average as g/animal/day. All animal experiment protocols were approved by Institutional Animal Ethics Committee of Connexios Life Sciences Pvt. Ltd.

### Oral glucose tolerance test (OGTT)

After 6 weeks of treatment OGTT was performed in 6 h fasted rats by orally administering CNX-011-67 (5 mg/kg) 30 min prior to 2 g/kg oral glucose load and blood samples collected from the tail vein −30 min before molecule administration and 0, 10, 20, 30, 60, 90 and 120 minutes after glucose load for glucose and insulin estimation. HOMA-beta and HOMA-IR were calculated to assess β-cell function and insulin action respectively.

### Prolonged post-prandial glucose profile

On day 42 of treatment, glucose in fed state was monitored at 2 h time intervals for a period of 8 h following the OGTT.

### Insulin tolerance test (ITT)

ITT was performed on day 48 in 6 h fasted rats. Blood samples, for glucose estimation, were collected prior to and at 2.5, 5, 10, 20, 30 and 60 min after administration of 2 IU/kg (i.p) insulin (Humulin R Insulin, Eli Lilly).

### Rat islet isolation

For in vitro experiments reported islets were isolated from male Wistar rats (8 weeks, 180-240 gm body weight; Charles River Lab, USA) as per previously reported [15] and size-matched purified islets were handpicked under a stereo-zoom microscope (Nikon, Japan).

### Insulin secretion and islet insulin content measurement

Stock fatty acid solutions were prepared by conjugating Palmitate with fatty acid free-BSA. In brief, Sodium palmitate was dissolved in 50% ethanol and heated at 60°C. The 500 mM stock thus prepared was conjugated with 10% BSA by stirring for 90 min to give a solution with a palmitate:BSA ratio of 3.6:1, filtered and used for experiments. Islets were cultured in growth medium (as control) or glucolipotoxic medium (16.7 mM glucose and 500 μM palmitate) either alone or with CNX-011-67 for 72 h and then transferred into 24-well plates containing 1 ml KRBH (2.5 mM glucose)/well, pre-incubated for 1 h and then induced with indicated glucose concentration alone or with 1 μM CNX-011-67 for 2 h. Secreted insulin was measured using an ELISA kit (Mercodia AB, Sweden) following the manufacturer’s protocol. Later, islet lysates were prepared in lysis buffer and used for measuring intracellular islet insulin content. Both secreted insulin and content were expressed as amount of insulin per islet.

### Measurement of ATP level

Rat islets were cultured in glucolipotoxic medium for 72 hrs, then incubated in KRBH buffer containing 2.5 mM glucose for 1 h and then induced with either 11 mM glucose (HG) alone or with 11 mM glucose and CNX-011-67 for 1 h. Islets were then lysed and ATP levels were estimated using ATP determination kit (Invitrogen) following the manufacturer’s protocol.

### RNA isolation, reverse transcription and quantitative real time polymerase chain reaction (qPCR)

Post 72 h of incubation, RNA was isolated, converted to cDNA (ABI, USA) and gene expression was measured using SYBR Green PCR Master Mix (Eurogenetic, Belgium). Gene primers were based on mRNA sequences from the GenBank nucleotide database and designed in-house. Genes analyzed in the present study were GCK, PDX1, Insulin, PPARa, TXNIP and CHOP. For quantification of gene expression, ?-actin was used as internal control (primer sequences are available upon request).

### Pancreas histology

Pancreas was fixed in 10% buffered neutral formalin for 48 h and paraffin-embedded, sectioned and stained with H&E or immunofluorescence stain for microscopic examination [16,17]. The pancreas section at the 25th, 26th, 27th , and 28th levels were taken for H&E, insulin immunofluorescence staining, Terminal deoxynucleotide transferase-mediated dUTP Nick end Labeling TUNEL) [18,19] and PDX1 and insulin double immunofluorescence labeling, respectively.

### Double immunofluorescence for insulin and PDX-1

Pancreatic sections were de-paraffinized and blocked in 1% BSA in PBS for 30 min at RT and incubated with a mixture of primary antibodies against insulin (Abexome, India) and PDX1 (Abcam USA) for 60 min at RT, followed by washing with PBS. Sections were then incubated with a mixture of secondary antibody (goat anti-mouse FITC conjugated secondary antibody for Insulin, Molecular Probes, Invitrogen and goat anti-rabbit conjugated with Alexafluor555, BioServices, India) for 30 min at RT and mounted using fluromount. Localization and distribution of insulin (green fluorescence) and PDX-1 (orange fluorescence) were assessed and the images were captured at 400X magnification. The number of PDX1-positive nuclei was counted in all islets (12 per animal) in the entire tissue section and values expressed as mean ± SEM.

### Assessment of islet apoptosis

To detect apoptosis, TUNEL staining was performed using ApopTag Peroxidase in situ Apoptosis detection kit (S7100) (Chemicon® International, Inc), according to the manufacturer’s instructions. The entire pancreas section was evaluated under light microscope at 400X magnification. Apoptosis-positive nuclei were counted within the islets on the whole pancreatic section area [19-21].

### Electron microscopy

Pancreas tissues were fixed in 3% glutaraldehyde for 24 h and processed for electron microscopy as described previously [17,22]. Three sections per group were scanned and images were captured using a Transmission electron microscope, Technai-G2 (Biotwin, FEI, Netherlands) at magnifications of 4800X, 11000X and 23000X. All photographs were analyzed manually. Insulin granules were assessed as mature granules (with an electron-dense core, tetrahedral or hexagonal in shape, surrounded by a peripheral halo) or as immature granules (with a less electron-dense core and a reduced or even absent halo) [23,24].

### Assessment of insulin signaling by AKT phosphorylation

Lean control, ZDF control and ZDF rats treated with CNX-011-67 for 7 weeks were sacrificed by cervical dislocation. 10 mg of tissue samples were collected from adipose (subcutaneous), muscle (soleus) and liver from each animal. Lysates were prepared by homogenization. Lysates (50 μg each from liver, muscle and adipose) were subjected to SDS-PAGE, transferred onto nitrocellulose membranes and probed with primary antibody against p-AKT and total AKT (Cell signaling, USA) and developed by enhanced chemiluminescence (West Pico, Thermo Scientific, USA). The relative levels of p-AKT compared to Total AKT were quantified using Image J Ver. 4.2, NIH, Bethesda.

### Statistical analysis

Statistical analyses were performed using Unpaired Student’s t-test or with One-Way ANOVA with Dunnett’s Post test correction, as appropriate. P value summary: (*) <0.05, (**) <0.01 and (***) <0.001 respectively, when compared with vehicle control.

## Results

### CNX-011-67 delays onset of fasting hyperglycemia in ZDF rats

**Figure 1:**
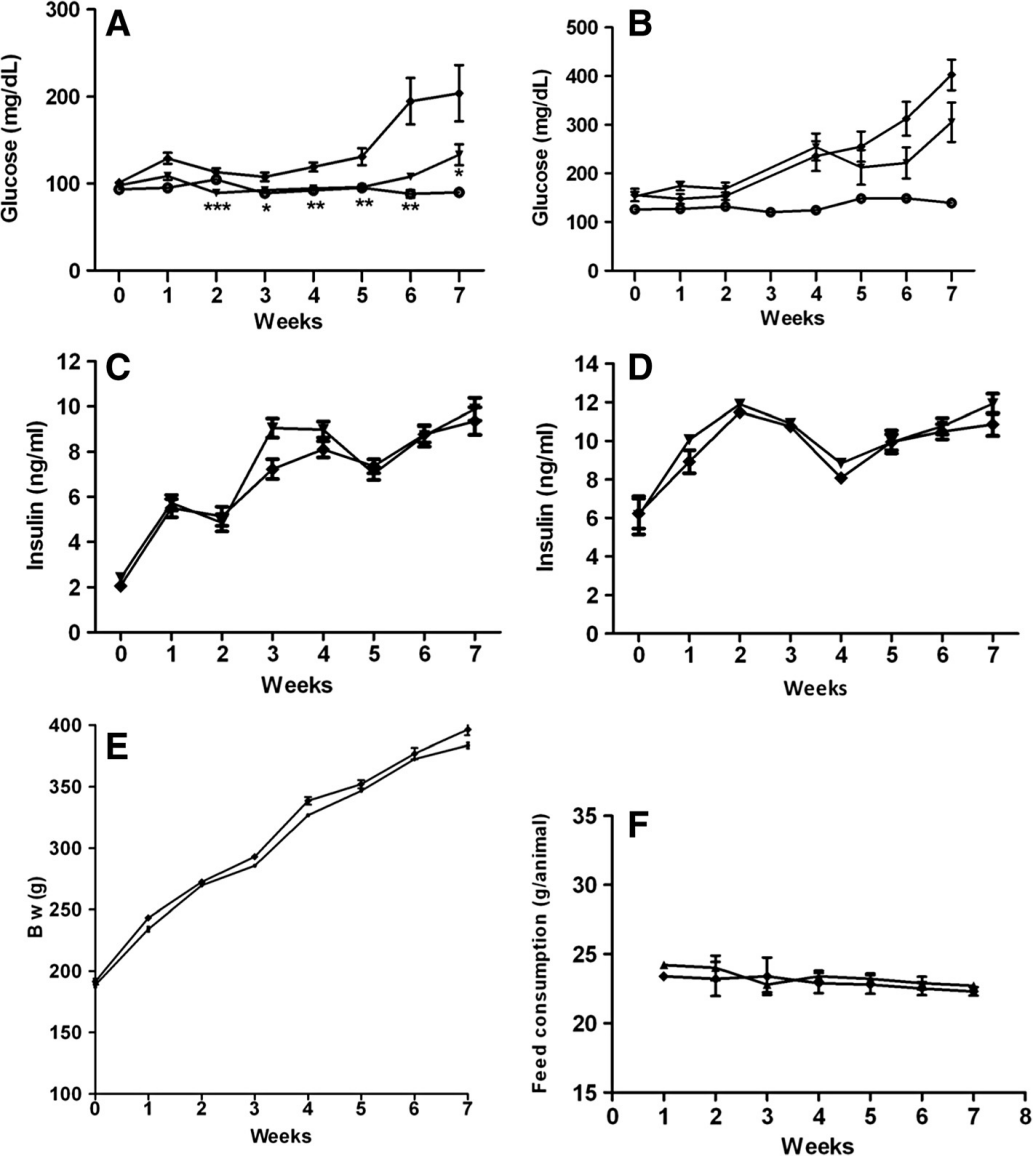
**Effects of CNX-011-67 on body weight, fed consumption, fasting and fed glucose and insulin levels.** Fasting (**A**) and fed (**B**) Glucose levels were monitored weekly. (Black circle)-Lean, (Black Diamond)-Vehicle control, (Black Triangle)-CNX-011-67 (5 mg/kg). Similarly, fasting (**C**) and fed (**D**) insulin levels were monitored weekly. (Black Diamond)-Vehicle control, (Black Triangle)-CNX-011-67 (5 mg/kg) Data in all panels are mean ± SEM (n = 10/group). (**E**) & (**F**) Body weight and feed consumption were monitored weekly. Statistical comparison between control and treatment group was conducted by One-way ANOVA followed by Dunnett’s post test correction for glucose while unpaired Student’s t test was performed for insulin levels. (*p < 0.05, **p < 0.01 and ***p < 0.001).

In lean control animals fasting glucose was 94 ± 4.3 mg/dl on day 0 and was 87 ± 1.7 mg/dl by end of the 7 week study. In the ZDF control animals fasting glucose reached 119 ± 5.14 mg/dl after 4 weeks treatment, kept increasing and reached 204 ± 32 mg/dl at end of 7 weeks. In contrast in the CNX-011-67 treated animals fasting glucose was 98 ± 2.7 mg/dl at start of the study and crossed the 100 mg/dl mark only after 6 weeks of treatment and reached 133 ± 12 at end of the study. Treatment with CNX-011-67 attenuated increase in fasting glucose and resulted in a 3 week delay in onset of hyperglycemia (Figure 1A).

### CNX-011-67 provides good control of hyperglycemia in ZDF rats

**Figure 2:**
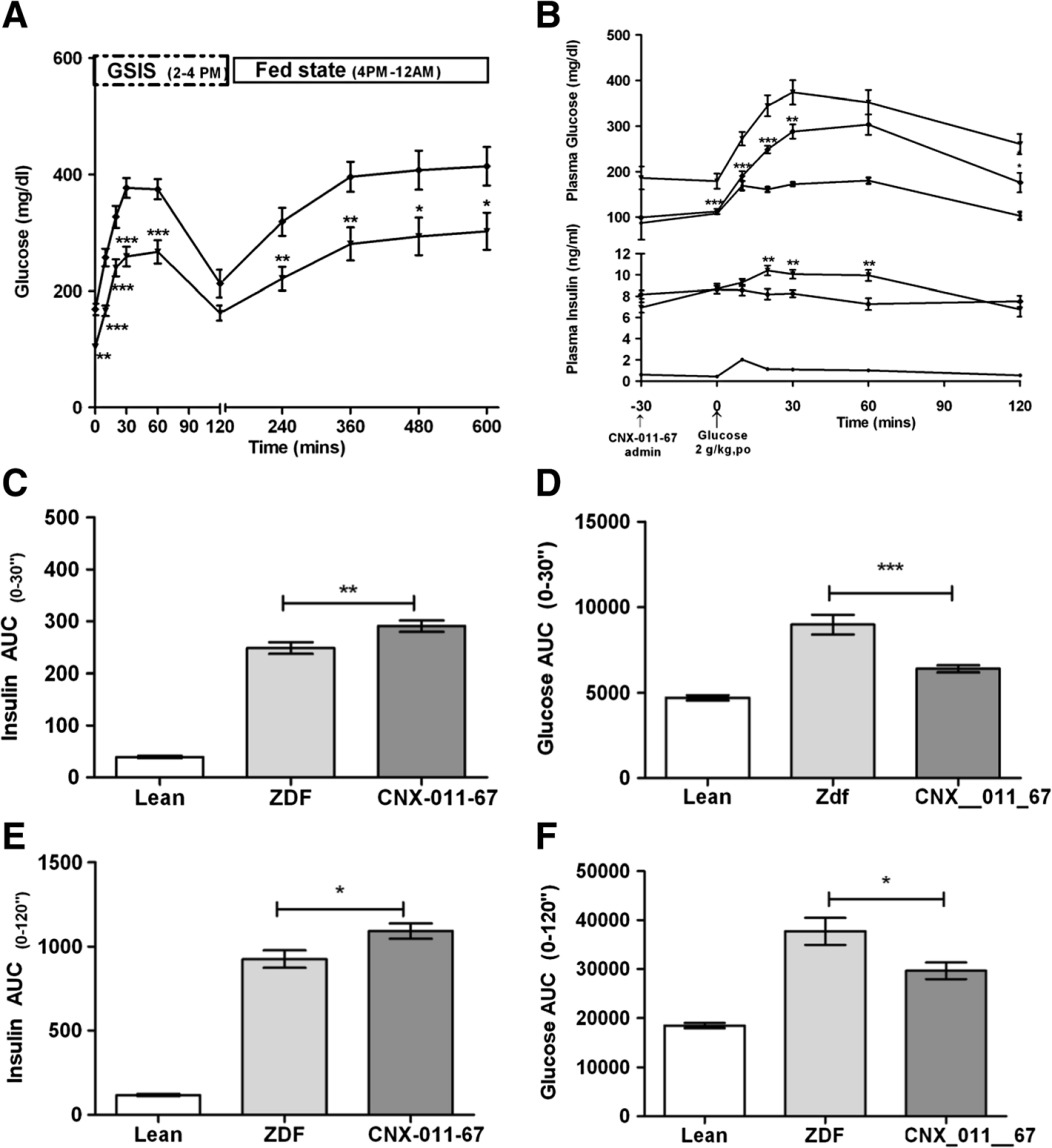
**Effects of CNX-011-67 treatment on blood glucose and insulin levels during glucose stimulated insulin secretion.** (**A**) A total of 8 h glucose profile was measured in fed state and Glucose AUC was calculated. (**B**) After oral administration of glucose (2 g/kg), plasma glucose and insulin were measured. Also Glucose (**D** &**F**) and Insulin (**C** &**E**) AUC were calculated. (Black circle)-Lean control, (Black Diamond)-Vehicle control and (Black Triangle)-CNX-011-67 (5 mg/kg)). Data in all panels are mean ± SEM. Data in all panels are mean ± SEM (n = 10/group). Statistical comparison was conducted by One-way ANOVA with Dunnett’s post test correction to arrive at the significance level when compared with ZDF control. (*p < 0.05, ** *p* <0.01 and *** *p* < 0.001).

In the control ZDF animals fed glucose levels increased from 155 ± 13 mg/dl on day 0 to 403 ± 31 mg/dl at end of 7 weeks study while in the CNX- 011–67 treated animals the corresponding levels for the study period were 152 ± 9.6 mg/dl to 305 ± 41 mg/dl respectively (Figure 1B). In the control ZDF animals blood glucose levels increased significantly upon restoration of access to food from 213 ± 23.79 mg/dl at the end of OGTT on week 6 to 414 ± 32.24 mg/dl at the end of 8 h follow up while in the CNX-011-67 treated animals the corresponding glucose levels were 162 ± 13 mg/dl and 303 ± 31.92 mg/dl respectively resulting in an 18% reduction in glucose AUC (Figure 2A). The food intake during the 8 h follow up period was however not quantified in either group.

### CNX-011-67 improves insulin secretion in ZDF rats

During the course of the study there was no difference in fasting and fed insulin levels between the ZDF control and CNX-011-67 treated animals (Figures 1C, D). However in an OGTT performed on day 42 of the study the 0–30’ insulin AUC was significantly higher (*p* < 0.05) (291 ± 11 vs. 249 ± 11) (Figure 2C) and the corresponding 0–30’ glucose AUC significantly lower (*p* < 0.01) (6389 ± 207 vs. 8964 ± 576) in the CNX-011-67 treated animals when compared to the ZDF control animals (Figure 2D). Similarly the 0–120’ insulin AUC was significantly higher (*p* < 0.05) (1093 ± 46 vs. 926 ± 52) (Figure 2E) and the corresponding 0–120’ glucose AUC significantly lower (*p* < 0.05) (29641 ± 1712 vs. 37716 ± 2767) in the CNX-011-67 treated animals (Figure 2F) when compared to the ZDF control animals indicating development of severe glucose intolerance in the control ZDF animals. In treated animals the increase in insulin levels from 6.95 ± 0.49 ng/ml at 0 min to 10.42 ± 0.47 ng/ml at 20 min and to 10.04 ± 0.42 ng/ml at 30 min post glucose load was highly significant (*p* < 0.01) (Figure 2B).

The significant difference in glucose tolerance in the OGTT observed with CNX-011-67 appears to be lost when the AUC Glucose is calculated above the basal values. This difference in the basal values between CNX-011-67 treated and control ZDF animals persists even after the onset of feeding upon switching off of the lights by 6 pm. Even though insulin levels at the various time points were not measured it appears that the difference in blood glucose levels could be due to enhanced insulin secretion in response to incremental glucose ingestion induced by CNX-011-67.

### CNX-011-67 improves insulin signaling in peripheral tissues and enhances glucose clearance

**Figure 3:**
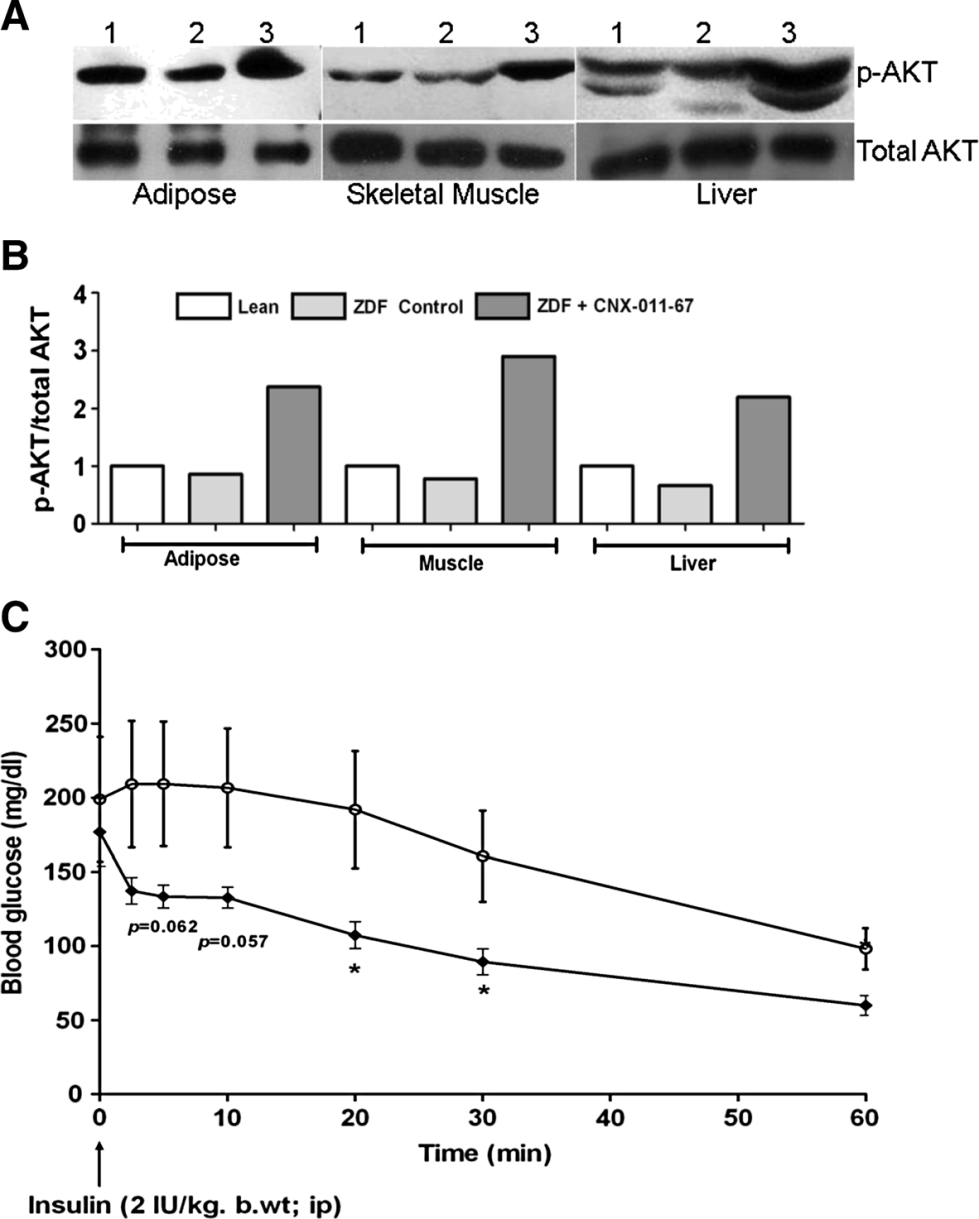
**Effects of CNX-011-67 treatment on insulin signaling in peripheral tissues.** (**A** and **B**) At the end of treatment, adipose, muscle and liver samples from lean (1), ZDF Control (2) and ZDF rats treated with CNX-011-67 (3) were subjected to western blots for p-AKT and total AKT. Blot in figure **A** is the data from one animal in each group (quantification was represented as fold change across all animals in each group, n = 5). (**C**) ITT was performed in male ZDF rats to determine the effect of CNX-011-67 on insulin induced glucose clearance. Glucose levels were monitored for a period of 60 minutes after insulin challenge. (Open circle)-Vehicle control (Black diamonds)-CNX- 011–67 (5 mg/kg). Data in all panels are mean ± SEM (n = 7 in control group / n = 8 in treated group). Statistical comparison between control and treatment group was conducted by unpaired Student’s t test for comparison with baseline values within groups (*p < 0.05, ** *p* < 0.01 and *** *p* < 0.001).

In ZDF control animals, an average decrease of 14, 22 and 34% in p-AKT to total-AKT levels was observed in adipose, skeletal muscle and liver respectively when compared to lean control group, indicating relatively higher insulin resistance. In contrast, the tissues from CNX-011-67 treated ZDF rats showed enhanced p-AKT to total-AKT levels (an average of 2.7, 3.7 and 3.3 fold increase in adipose, skeletal muscle and liver, respectively) over those from ZDF control animals (Figure 3B) indicating an improvement in insulin signaling in the periphery. The representative blot from one animal in each group has been presented in Figure 3A.

In insulin tolerance test the decrease in blood glucose levels in the CNX-011-67 treated animals, non-significant in the first 10 min, became significant (*p* < 0.05) at 20 and 30 min post insulin administration when compared with control ZDF animals (Figure 3C). This agrees with the significant difference observed in HOMA-IR between ZDF control animals (64 ± 6.17) and CNX-011-67 treated animals (42 ± 6) indicating a reduction in insulin resistance in the treated animals (Table 1).

**Table 1:** Effects of CNX-011-67 on blood HbA1c, Fructosamine, PDX1 and apoptotic positive cells

**Parameters**		** Groupings**	
	**Lean control**	**ZDF control**	**CNX-011-67 (5 mg/kg)**
HbA1c	3.84 ± 0.08	5.50 ± 0.30	5.18 ± 0.11
Fructosamine	92.47 ± 10.68	236.36 ± 19.08	111.25 ± 10.61**
HOMA-IR	2.16 ± 0.74	64.26 ± 6.18	41.82 ± 2.59*
HOMA-beta	117.34 ± 39.35	1520.20 ± 227.30	2206.79 ± 418.69
PDX1 positive cells	650 ± 49	461 ± 47.50	589 ± 43
Apoptotic cells	00 ± 00	47 ± 8.7	29 ± 4.6

Data in all panels are mean ± SEM (n = 10/group). Statistical comparison was conducted by One-way ANOVA with Dunnett’s post test correction to arrive at the significance level when compared with ZDF control. (*p < 0.05 and **p < 0.01).

### CNX-011-67 decreases serum FFA and triglyceride levels in ZDF rats

**Figure 4:**
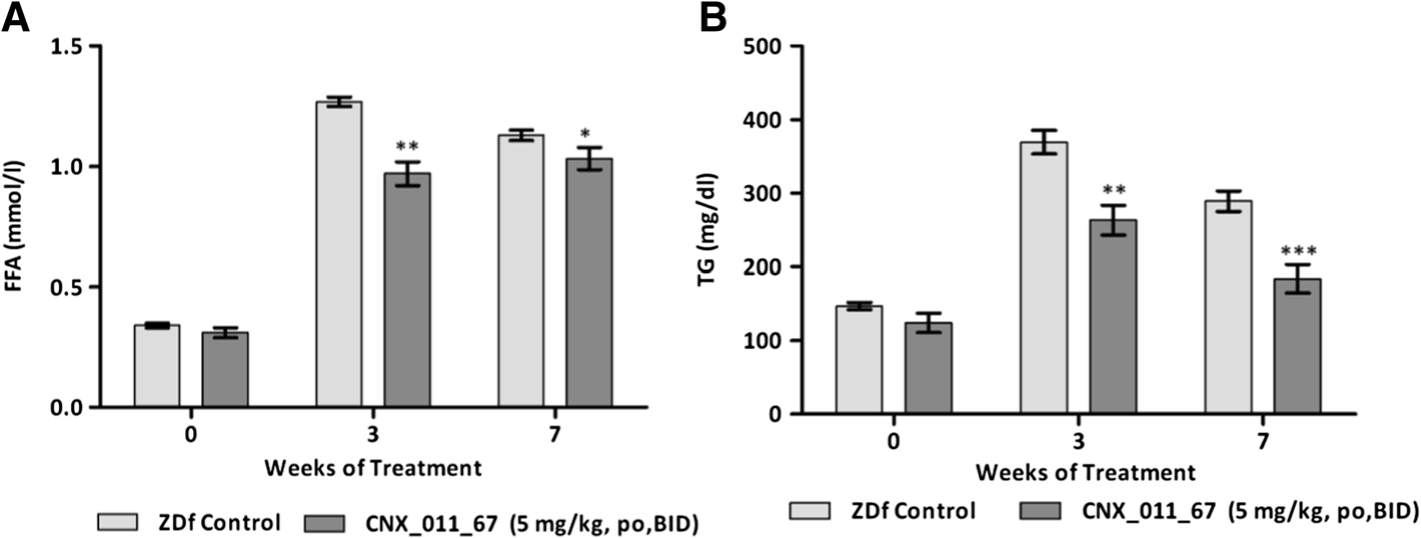
**Effects of CNX-011-67 on FFA and TG levels in male ZDF rats.** CNX-011-67 reduced both serum FFA (**A**) and serum TG (**B**) in ZDF rats when compared with vehicle control rats. Data in all panels are mean ± SEM (*n* = 10/group). Statistical comparison between control and treatment group was conducted by unpaired Student’s t test for comparison with baseline values within groups (*p < 0.05, ** *p* < 0.01 and ***p < 0.001).

At the end of 3rd week there was a significant (*p* < 0.01) decrease in both plasma FFAs (0.97 ± 0.05 mmol/l vs. 1.27 ± 0.02 mmol/l) and triglyceride levels (263 ± 20 mg/dl vs. 370 ± 16 mg/dl) in the CNX-011-67 treated animals when compared to the ZDF control animals. Similar differences were observed even at end of the study between ZDF control and treated animals (Figures 4A, B).

### CNX-011-67 decreases HbA1c and fructosamine

After 7 weeks of treatment HbA1c level was 5.18% in CNX-011-67 compared to 5.5% in the control ZDF group and the difference was not statistically significant. However plasma fructosamine levels of 111 ± 26 μmol/l in CNX-011-67 treated animals was significantly (*p* < 0.01) lesser than 237 ± 17 μmol/l in the control ZDF animals (Table 1).

### CNX-011-67 significantly improves insulin secretion in rat pancreatic islets

**Figure 5:**
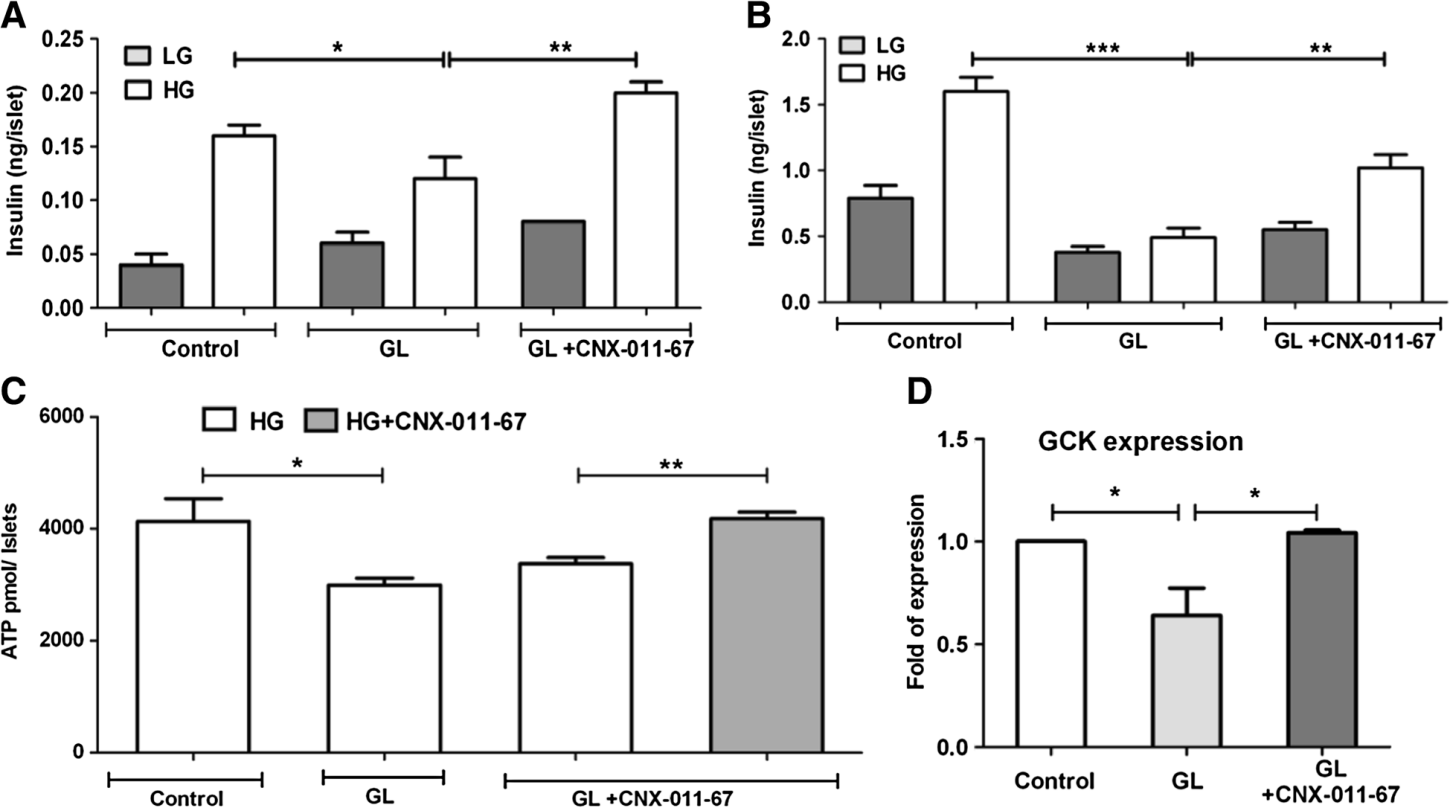
**Effect of CNX-011-67 on insulin secretion, content, ATP and GCK expression.** Rat islets were cultured under normal condition (control) or with high glucose and palmitate (glucolipotoxic condition; GL) or with CNX-011-67 under GL condition for 72 h. Post 72 h, islets was treated with 2 mM glucose (LG) or 11 mM glucose (HG) for 2 h and secreted insulin was measured (**A**). Islets were then lysed and lysate was used for intracellular insulin content measurement (**B**). Data (A) and (B) are representative of ten independent experiments (N = 10, *n* = 4). For ATP measurements, post 72 h of glucolipotoxicity in the presence or absence of CNX-011-67, islets were incubated with either 11 mM glucose (HG) alone or with HG + CNX-011-67 for 1 h. Islets were then lysed and ATP levels were estimated (N = 1, *n* = 3) (**C**). For GCK expression studies (**D**), RNA was isolated post 72 h of treatment and expression of GCK mRNA was measured as described under Methods (N = 2, *n* = 4). Statistical analysis was performed by Students t-test (Two-way, unpaired) or One-Way ANOVA with Dunnett’s post test correction, as appropriate (*p < 0.05, ** *p* < 0.01 and ***p < 0.001).

Exposure of islets to chronic glucolipotoxic conditions significantly (*p* < 0.05) reduced glucose stimulated insulin secretion. However, treatment with CNX-011-67 during exposure to glucolipotoxicity significantly (*p* < 0.01) restored GSIS (Figure 5A). Similarly treatment with CNX-011-67 significantly increased (*p* < 0.01) islet insulin content when compared to that in chronic glucolipotoxic control islets (Figure 5B).

### CNX-011-67 enhances glucose metabolism and ATP synthesis

Glucose-mediated increase in ATP levels and mRNA expression of GCK were significantly reduced under glucolipotoxic conditions as compared to control islets. Chronic activation by CNX-011-67 reversed the effect of glucolipotoxicity and significantly enhanced islet ATP content and expression of GCK mRNA (Figures 5C, D).

### CNX-011-67 enhances mRNA expression of genes involved in insulin synthesis, and reduces expression of genes involved in stress and apoptosis

**Figure 6:**
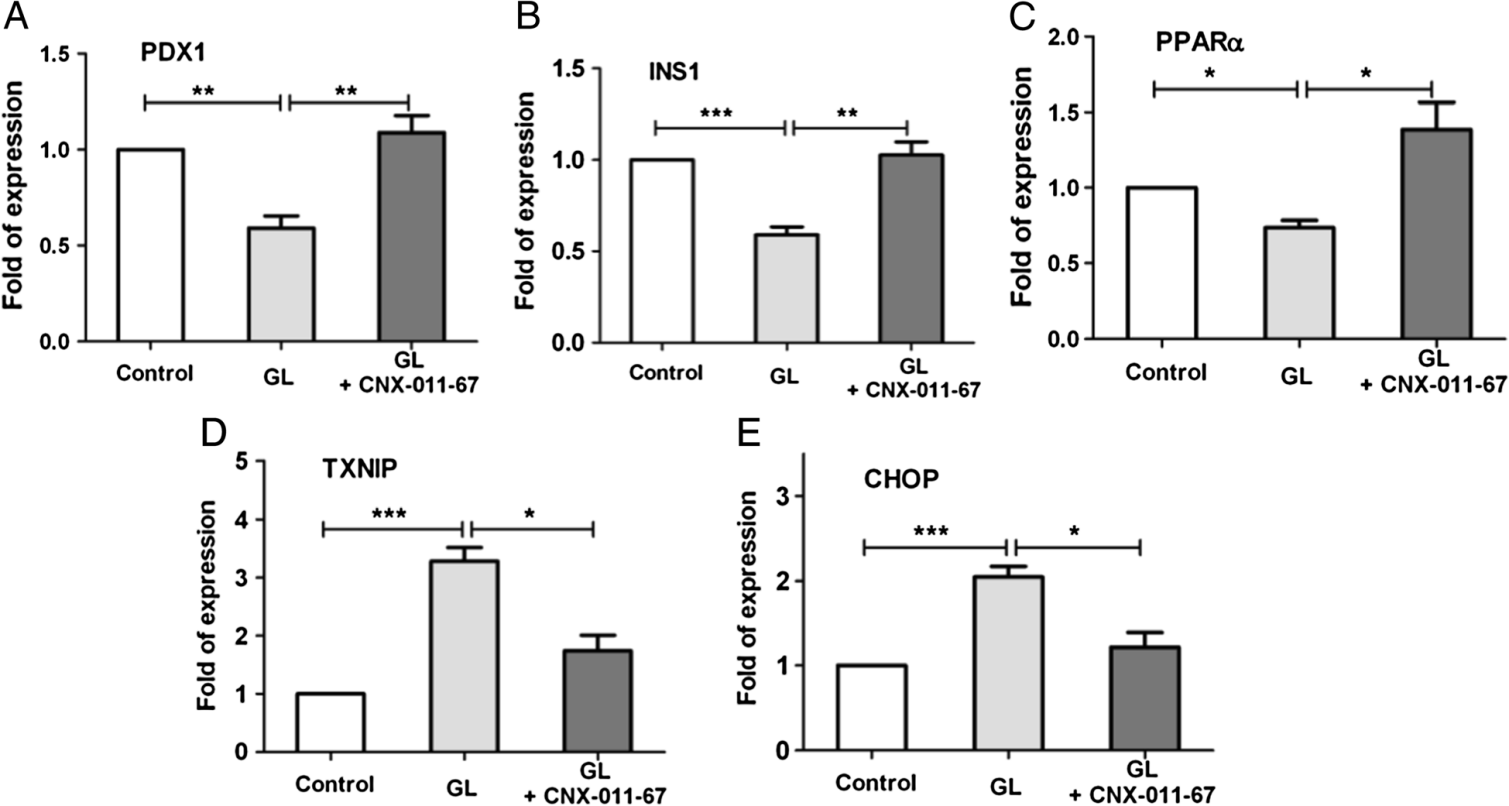
**Impact of CNX-011-67 on islet insulin synthesis and β-cell stress.** Post 72 h of treatments, RNA was isolated and expression of (**A**) PDX1, (**B**) INS1, (**C**) PPARα, (**D**) TXNIP and (**E**) CHOP mRNA expression were measured as described under Methods (N = 2, *n* = 4). Statistical analysis was performed by One-Way ANOVA with Dunnett’s post test correction (*p < 0.05, **p < 0.01 and ***p < 0.001).

Chronic glucolipotoxicity reduced islet mRNA expression of PDX1, INS1 and PPARa whereas increased TXNIP and CHOP significantly when compared to control islets. Exposure of islets to CNX-011-67 under chronic glucolipotoxic conditions, significantly restored expression of these same genes to wild type levels (Figures 6A, B, C, D, E).

### CNX-011-67 improves PDX1 expression in islets

**Figure 7:**
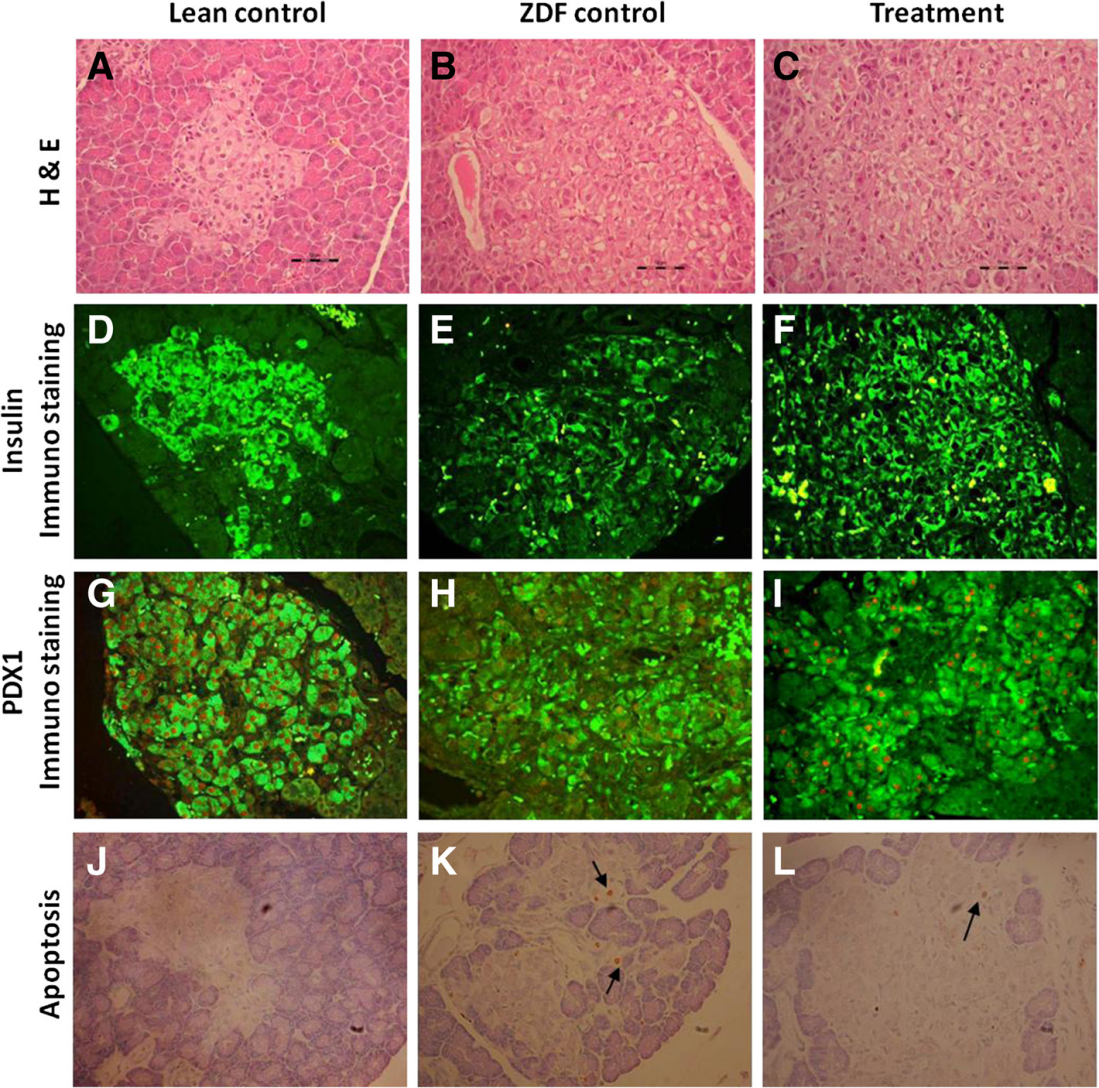
**Histology, Immunohistochemistry of Pancreas and metabolic parameters in male ZDF rats. A-C**: **H** and **E** stained pancreatic Islets sections from Lean control, ZDF control and CNX-011-67 treatment group. Magnifications at 400X. Islets with normal architecture from Lean control (**A**,**B**) and with distorted architecture in ZDF animals **D-F**: Immunofluorescent staining for insulin in consecutive sections of Pancreas. Immunostaining intensity of B-cells for Insulin is good in Lean control, poor in ZDF control and improved in Treatment group. **G-I**: Double immuno fluorescence staining for PDX1 (red) and insulin (green) of rat pancreatic islets. Compared to ZDF control the number of PDX1 positive nucleus (red) in the treatment appears to be increased. **J-L**: Brown colored apoptotic beta cells (arrow) in a pancreatic islet. Compared to ZDF control, apoptotic beta cells in treatment are found to be reduced.

Beta cells in the ZDF lean controls displayed good immuno-reactivity to PDX1 antibody and high staining intensity. Male ZDF animals with distorted islet architecture showed uneven immunoreactivity to PDX1 antibody and staining intensity was either low or completely absent. CNX-011-67 treated animals showed a marked improvement in islet morphology and displayed intense PDX1 immunostaining in comparison with ZDF control animals. A reduction in PDX1-positive nuclei number was observed in ZDF animals (461 ± 48 ZDF vs. 650 ± 49, lean control), whereas a marked improvement was observed in CNX-011-67 treated animals (589 ± 43 vs. 461 ± 48, ZDF control) (Figures 7G, I). The values, though not statistically significant, suggest enhanced PDX-1 expression in CNX-011-67 treated animals.

### CNX-011-67 decreases β-Cell apoptosis

Morphological features of apoptosis, including pyknotic nuclei, were readily detectable in all pancreatic sections. The number of TUNEL-positive apoptotic beta cells was markedly increased in ZDF control animals compared with lean control. There were no fragmented nuclear apoptotic cells localized within the islets in lean control animals. The number of TUNEL positive apoptotic cells were significantly reduced in CNX-011-67 treated animals (47 ± 9 vs. 29 ± 5). A reduction of ~35% in apoptosis-positive nuclei was observed upon treatment with CNX-011-67 (Table 1).

### Electron microscopy

**Figure 8:**
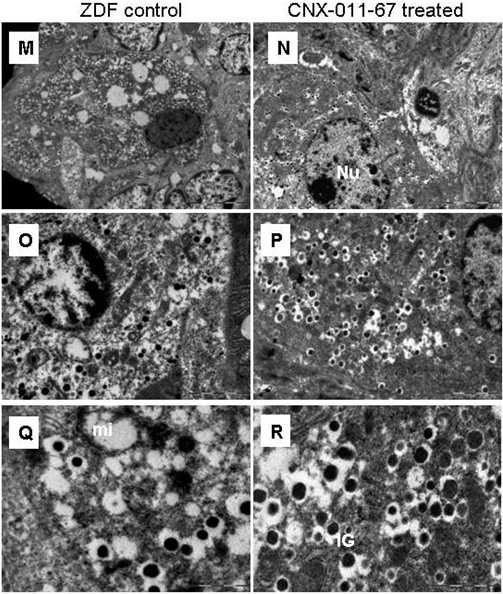
**Electron microscope images of Beta cells from male ZDF and CNX-011-67 treated rats.** Magnification at 4800 X (**M** and **N**), 11000 X (**O** and **P**), 23000X (**Q** and **R**). NU- Nucleus, mi-Mitochondria, IG- Insulin granules.

Islets from ZDF control animals were either without or with a decreased number of insulin granules with electron-dense cores with many situated away from the plasma membrane indicating low number of docked granules ready for release (Figure 8). These islets also had large number of vacuoles. Islets from CNX-011-67 treated animals displayed an appreciable improvement in number of insulin granules with electron-dense cores surrounded by peripheral halo, tetrahedral or hexagonal in shape well distributed within the cytoplasm and also arrayed close to the plasma membrane indicating much improved docking of vesicles.

### CNX-011-67 improves islet insulin content and morphology

Immunohistochemical analysis showed improvement in insulin stained area (morphological assessment) and a larger number of insulin stained cells in CNX-011-67 treated animals (694 ± 114 vs. 526 ± 26, ZDF control) indicating improved insulin content when compared to control ZDF animals (Figures 7D, E, F). Islets from ZDF lean control animals were compact and had a typical organized architecture while islets from control ZDF animals appeared enlarged and disorganized indicating loss of architecture. H&E staining of islets from ZDF control animals indicated a distorted architecture of islets which was distinctly improved upon CNX-011-67 treatment (Figures 7A, B, C). Enlarged and disorganized islets with extensions into the surrounding exocrine tissue were observed in ZDF control islets and a marked improvement was observed in animals treated with CNX-011-67 for seven weeks.

## Discussion

A significant finding of the study has been the robust control of both the onset and progress of hyperglycemia by CNX-011-67. Considering that fed and fasting insulin levels did not vary between the animals in the treated and the control groups during the study period it appears that the enhanced first and second phase insulin secretion induced by the agonist (Figure 2B) is responsible for the robust glycemic control achieved in the treated animals. It is well documented in literature that an enhanced early phase insulin secretion has a direct effect on shutting off hepatic glucose output [25,26] and insulin is known to suppress lipolysis in adipocytes [27].

CNX-011-67 does not display any off target effect on lipolysis in 3 T3-L1 cells (data not shown) nor does the molecule activate PPARγ at concentrations below 10 μM. Though data on lipolysis in adipocytes derived from control and CNX-011-67 treated animals from this study is not available the enhanced level of p-Akt in the adipocytes from CNX-011-67 treated animals (Figures 3A & B) seems to indicate enhanced insulin signalling even at the end of the study period. We therefore suggest that the reduction in serum free fatty acids level in the treated rats is a direct effect of the enhanced insulin delivery and action in the adipocytes that reduces lipolysis. We are aware that additional studies are necessary to establish the direct effect of CNX-011-67 mediated increase in first phase insulin secretion in controlling lipolysis in adipocytes. The enhanced first phase insulin secretion (Figure 2B), reduction in free fatty acids (Figure 4A) and the increased insulin signaling in liver observed in CNX-011-67 treated animals (Figure 3A) perhaps explain the significant control over fasting glucose achieved in the study.

Even though there was no reduction in food intake or in body weight all through the treatment period the significant attenuation of the increase in blood glucose levels in the initial phase of the feeding period (6 pm – 12 pm; Figure 2A) is indicative of reduction in peripheral insulin resistance in CNX-011-67 treated animals. The reduction in insulin resistance assumes importance as CNX-011-67 does not impact GLP1 levels in vivo (14 ± 0.58 vs. 15.98 ± 0.32 ZDF control active GLP1).

A reduction in circulating free fatty acid levels is reported to impact functioning of various organs and can thus affect multiple physiological parameters. Increased flux of free fatty acid into muscle reduces glucose uptake, into liver enhances gluconeogenesis and into pancreas can reduce insulin secretion and increase apoptosis [28]. The enhanced insulin secretion in response to glucose load (Figure 2B) and the significant reduction in free fatty acid levels early in treatment (Figure 4A) could have facilitated increased insulin signaling in liver, adipose and muscle (Figure 3A) thereby resulting in good control of fed glucose levels (Figure 2A). It is tempting to speculate (in absence of any supporting data) that the significant reduction in serum triglycerides could be due to a reduction in lipolysis in adipocytes (and consequent reduction in flux of free fatty acids into liver) and an improved glucose uptake in muscle.

In the present study it can perhaps be assumed that the significant control on serum free fatty acids levels achieved during the initial weeks of treatment has to a significant extent reduced insulin resistance in the periphery and thus improved glycemic control. In fact there is a significant reduction in HOMA-IR at end of the study (Table 1) and a significant increase in insulin secretion in response to glucose on day 42 of treatment when compared to control ZDF rats (Figure 2A). The insulin tolerance test (Figure 4) indicates overall reduction in insulin resistance in CNX-011-67 treated rats.

Multiple genes are involved in maintaining beta cell function. We restricted our study to a few select genes namely PDX1, INS1, GCK, PPARα, CHOP and TXNIP known to have significant impact on beta cell glucose metabolism, insulin synthesis, secretion and apoptosis.

Peroxisome proliferator-activated receptor alpha (PPARalpha) is known to modulate insulin secretion [29] and is reported to protect against fatty acid induced dysfunction by preserving carbohydrate metabolism [30] and can also increase insulin gene transcription. TXNIP serves as a critical link between glucose toxicity and beta-cell apoptosis [31], mediates ER stress [32] and glucocorticoid mediated [33] cell death and lack of TXNIP protects against mitochondria-mediated apoptosis [34].

We consider the enhanced insulin-stained area in islets to be probably due to CNX-011-67 mediated activation of GPR40 that can potentially stimulate ATP synthesis and modulate expression of genes mentioned above under the prevailing conditions of FFAs and glucose levels in the animals. Data from OGTT (Figure 2B), immunohistochemistry (Figures 7A, B, C), TUNEL assay (Figures 7J, K, L) and the increase in number of insulin granules with electron dense cores (Figure 8) seem to strongly support the above assumption.

Significant differences between control ZDF and CNX-011-67 treated animals in fed glucose levels (255 ± 31 vs. 212 ± 35 mg/dl) developed only by week 5 and continued to widen till end of study (406 ± 31 vs. 305 ± 41 mg/dl) while moderate differences in fasting glucose levels (119 ± 5.1 vs. 94.3 ± 4 mg/dl) that developed by week 4 of treatment sharply increased by week 6 (195 ± 26 vs. 108.3 ± 3.7 mg/dl) and week 7 (204 ± 32 vs. 133 ± 12 mg/dl). The HbA1c reduction of 0.32% reflects the control on glucose exercised by CNX-011-67 mainly during this period. The half-lives of glycosylated Hb being 30, 16 and 15 days in man, mouse and rat, respectively [35] the difference in HbA1c levels of 0.32%, between control ZDF and CNX-011-67 treated animals is achieved in a time period that covers two half-life of rat glycosylated Hb. The significant difference in fructosamine levels indicates effective control of glucose levels by CNX-011-67. Inspite of an improvement in first phase insulin secretion and reduction in fasting glucose levels beta cell function as assessed by HOMA-B displayed non-significant improvement. It is reported that estimates of cell function using HOMA have been shown to correlate well with estimates using hyperglycemic clamps and with the acute insulin response from the IVGTT [36]. In this study we have not employed either hyperglycemic clamps or performed IVGTT and hence the HOMA-B arrived at may not reflect the actual improvement in beta cell function achieved in this study.

In male ZDF rats a combination of lipotoxicity and hyperglycemia increase loss of β-cells through apoptosis [37,38] and peripheral acting agents like Rosiglitazone, Metformin, Troglitazone or even the DPP-IV inhibitor FE 999011 that provided significant control over free fatty acids, glucose and triglycerides, improved beta cell function and delayed the progression to diabetes [3,39,40]. Some of the pharmacological effects shown by CNX-011-67 in the present study are similar to the effects observed earlier in preclinical studies with other GPR40 agonists [7,8].

## Conclusions

The delay in the onset and robust control of hyperglycemia observed in this study appear to be mediated by the combined effects of enhanced phasic insulin secretion, improvement in glucose sensitivity of beta cells, increased insulin content and reduction in beta cell apoptosis. Though we have not determined the changes in gene expression in the beta cells of treated rats in this study the increase in expression of PDX1 protein and increase in insulin stained area (Figure 7) and a reduction in beta cell apoptosis suggest that CNX-011-67 treatment could have modulated expression of many of the critical genes involved in insulin synthesis, insulin secretion and beta cell apoptosis in the male ZDF rats. From the foregoing data it appears that CNX-011-67 has the potential to provide good and durable glycemic control in type 2 diabetes patients.

## Competing interests

The authors declare that they have no competing interests.

## Authors’ contributions

NG, JS, SB, DNVR, MNL, PCS, VS, AR carried out all the experiments; AD, MKS, KA, MKV, YM designed the study, carried out the analysis. MOA, MVV and BPS interpreted the data and drafted the manuscript. MRJ supervised the progress and critically revised the manuscript. All authors read and approved the final manuscript.

## Pre-publication history

The pre-publication history for this paper can be accessed here:

http://www.biomedcentral.com/2050-6511/14/28/prepub

## Supplementary Material

Additional file 1: Figure S1 CNX-011-67 is highly specific to GPR40. Click here for file

## References

[B1] Fineman MS Cirincione BB Maggs D Diamant M GLP-1 based therapies: differential effects on fasting and postprandial glucose Diabetes Obes Metab 2012 14 675 688 10.1111/j.1463-1326.2012.01560.x 22233527

[B2] Richter B Bandeira-Echtler E Bergerhoff K Clar C Ebrahim SH Pioglitazone for type 2 diabetes mellitus Cochrane Database Syst Rev 2006 18 CD006060 10.1002/14651858.CD006060.pub2 PMC8991699 17054272

[B3] Sreenan S Sturis J Pugh W Burant CF Polonsky KS Prevention of hyperglycemia in the Zucker diabetic fatty rat by treatment with metformin or troglitazone Am J Physiol 1996 271 742 747 10.1152/ajpendo.1996.271.4.E742 8897863

[B4] Briscoe CP Peat AJ McKeown SC Corbett DF Goetz AS Littleton TR McCoy DC Kenakin TP Andrews JL Ammala C Fornwald JA Ignar DM Jenkinson S Pharmacological regulation of insulin secretion in MIN6 cells through the fatty acid receptor GPR40: identification of agonist and antagonist small molecules Br J Pharmacol 2009 148 619 628 16702987 10.1038/sj.bjp.0706770 PMC1751878

[B5] Shapiro H Shachar S Sekler I Hershfinkel M Walker MD Role of GPR40 in fatty acid action on the beta cell line INS-1E Biochem Biophys Res Commun 2005 335 97 104 10.1016/j.bbrc.2005.07.042 16081037

[B6] Fujiwara K Maekawa F Yada T Oleic acid interacts with GPR40 to induce Ca2+ signaling in rat islet beta-cells: mediation by PLC and L-type Ca2+ channel and link to insulin release Am J Physiol Endocrinol Metab 2005 289 670 677 10.1152/ajpendo.00035.2005 15914509

[B7] Tan CP Feng Y Zhou YP Eiermann GJ Petrov A Zhou C Lin S Salituro G Meinke P Mosley R Akiyama TE Einstein M Kumar S Berger JP Mills SG Thornberry NA Yang L Howard AD Selective small-molecule agonists of G protein-coupled receptor 40 promote glucose-dependent insulin secretion and reduce blood glucose in mice Diabetes 2008 57 2211 2219 10.2337/db08-0130 18477808 PMC2494688

[B8] Tsujihata Y Ito R Suzuki M Harada A Negoro N Yasuma T Momose Y Takeuchi K TAK-875, an orally available G protein-coupled receptor 40/free fatty acid receptor 1 agonist, enhances glucose-dependent insulin secretion and improves both postprandial and fasting hyperglycemia in type 2 diabetic rats J Pharmacol Exp Ther 2011 339 228 327 10.1124/jpet.111.183772 21752941

[B9] Lin DC Zhang J Zhuang R Li F Nguyen K Chen M Tran T Lopez E Lu JY Li XN Tang L Tonn GR Swaminath G Reagan JD Chen JL Tian H Lin YJ Houze JB Luo J AMG 837: a novel GPR40/FFA1 agonist that enhances insulin secretion and lowers glucose levels in rodents PLoS One 2011 6 e27270 10.1371/journal.pone.0027270 22087278 PMC3210765

[B10] Luo J Swaminath G Brown SP Zhang J Guo Q Chen M Nguyen K Tran T Miao L Dransfield PJ Vimolratana M Houze JB Wong S Toteva M Shan B Li F Zhuang R Lin DC A Potent class of GPR40 full agonists engages the enteroinsular axis to promote glucose control in rodents PLoS One 2012 7 e46300 10.1371/journal.pone.0046300 23056280 PMC3467217

[B11] Burant CF Viswanathan P Marcinak J Cao C Vakilynejad M Xie B Leifke E TAK-875 versus placebo or glimepiride in type 2 diabetes mellitus: a phase 2, randomised, double-blind, placebo-controlled trial Lancet 2012 379 1403 1411 10.1016/S0140-6736(11)61879-5 22374408

[B12] Kaku K Araki T Yoshinaka R Randomized, Double-Blind, Dose-Ranging Study of TAK-875, a Novel GPR40 Agonist, in Japanese Patients With Inadequately Controlled Type 2 Diabetes Diabetes Care 2013 36 245 250 10.2337/dc12-0872 23086138 PMC3554318

[B13] Tokuyama Y Sturis J DePaoli AM Takeda J Stoffel M Tang J Sun X Polonsky KS Bell GI Evolution of beta cell dysfunction in the male Zucker diabetic fatty rat Diabetes 1995 44 1447 1457 10.2337/diabetes.44.12.1447 7589853

[B14] Yuen VG Vera E Battell ML Li WM McNeill JH Acute and chronic oral administration of bis(maltolato) oxovanadium (IV) in Zucker diabetic fatty (ZDF) rats Diabetes Res Clin Pract 1999 43 9 19 10.1016/S0168-8227(98)00120-X 10199584

[B15] Carter JD Dula SB Corbin KL Wu R Nunemaker CS A practical guide to rodent islet isolation and assessment Biol Proced Online 2009 11 3 31 10.1007/s12575-009-9021-0 19957062 PMC3056052

[B16] Luna L AFIP Manual of Histological Staining Methods 1968 New York: McGraw Hill

[B17] Culling CFA Allison RT Bair WT Cellular pathology technique 1985 London: Butterworths

[B18] Kerr JFR Wyllie AH Currie AR Apoptosis: a basic biological phenomenon with wideranging implications in tissue kinetics Br J Cancer 1972 26 239 257 10.1038/bjc.1972.33 4561027 PMC2008650

[B19] Wyllie AH Kerr JFR Currie AR Cell death: the significance of apoptosis Int Rev Cytol 1980 68 251 306 7014501 10.1016/s0074-7696(08)62312-8

[B20] Garrity MM Burgart LJ Riehle DL Hill EM Sebo TJ Witzig T Identifying and Quantifying Apoptosis: Navigating Technical Pitfalls Mod Pathol 2003 16 389 394 10.1097/01.MP.0000062657.30170.92 12692204

[B21] Bruin JE Gerstein HC Morrison KM Holloway AC Increased pancreatic beta cell apoptosis following fetal and neonatal exposure to nicotine is mediated via the mitochondria Toxicol Sci 2008 103 362 370 10.1093/toxsci/kfn012 18203686

[B22] Watari N Tsukagoshi N Honma Y The correlative light and electron microscopy of the islets of Langerhans in some lower vertebrates Arch Histol 1970 31 371 392 10.1679/aohc1950.31.371 4194556

[B23] Mihail N Craciun C An ultrastructural description of the cell types in the endocrine pancreas of the pigeon Anat Anz Jena 1982 152 229 237 6760744

[B24] Misawa E Tanaka M Nomaguchi K Nomaguchia K Yamada M Toida T Takase M Iwatsuki K Kawada T Administration of phytosterols isolated from aloe vera gel reduce visceral fat mass and improve hyperglycemia in Zucker diabetic fatty (zdf) rats Obes Res Clin Pract 2008 2 239 245 10.1016/j.orcp.2008.06.002 24351850

[B25] Del Prato S Loss of early insulin secretion leads to postprandial hyperglycaemia Diabetologia 2003 46 2 8 10.1007/s00125-002-0930-6 12652352

[B26] Rebrin K Steil GM Getty L Bergman RN Free fatty acid as a link in the regulation of hepatic glucose output by peripheral insulin Diabetes 1995 44 1038 1045 10.2337/diabetes.44.9.1038 7657026

[B27] Koopmans SJ Sips HC Bosman J Radder JK Krans HM Antilipolytic action of insulin in adipocytes from starved and diabetic rats during adenosine-controlled incubations Endocrinology 1989 125 3044 3050 10.1210/endo-125-6-3044 2684615

[B28] Bajaj M Suraamornkul S Romanelli A Cline GW Mandarino LJ Shulman GI DeFronzo RA Effect of a sustained reduction in plasma free fatty acid concentration on intramuscular long-chain fatty Acyl-CoAs and insulin action in type 2 diabetic patients Diabetes 2005 54 3148 3153 10.2337/diabetes.54.11.3148 16249438

[B29] Sugden MC Holness MJ Potential role of peroxisome proliferator-activated receptor-alpha in the modulation of glucose-stimulated insulin secretion Diabetes 2004 53 Suppl 1 S71 S81 14749269 10.2337/diabetes.53.2007.s71

[B30] Frigerio F Brun T Bartley C Usardi A Bosco D Ravnskjaer K Mandrup S Maechler P Peroxisome proliferator-activated receptor alpha (PPARalpha) protects against oleate-induced INS-1E beta cell dysfunction by preserving carbohydrate metabolism Diabetologia 2010 53 331 340 10.1007/s00125-009-1590-6 19908022

[B31] Chen J Saxena G Mungrue IN Lusis AJ Shalev A Thioredoxin-interacting protein: a critical link between glucose toxicity and beta-cell apoptosis Diabetes 2008 57 938 944 10.2337/db07-0715 18171713 PMC3618659

[B32] Oslowski CM Hara T O’Sullivan-Murphy B Kanekura K Lu S Hara M Ishigaki S Zhu LJ Hayashi E Hui ST Greiner D Kaufman RJ Bortell R Urano F Thioredoxin-interacting protein mediates ER stress-induced β cell death through initiation of the inflammasome Cell Metab 2012 16 265 273 10.1016/j.cmet.2012.07.005 22883234 PMC3418541

[B33] Reich E Tamary A Sionov RV Melloul D Involvement of thioredoxin-interacting protein (TXNIP) in glucocorticoid-mediated beta cell death Diabetologia 2012 55 1048 1057 10.1007/s00125-011-2422-z 22246375

[B34] Chen J Fontes G Saxena G Poitout V Shalev A Lack of TXNIP protects against mitochondria-mediated apoptosis but not against fatty acid-induced ER stress-mediated beta-cell death Diabetes 2010 59 440 447 10.2337/db09-0949 19875615 PMC2809961

[B35] Rendell M Stephen PM Paulsen R Valentine JL Rasbold K Hestorff T Eastberg S Shint DC An interspecies comparison of normal levels of glycosylated hemoglobin and glycosylated albumin Comp Biochem Physiol B 1985 81 819 822 4042626 10.1016/0305-0491(85)90072-0

[B36] Wallace TM Levy JC Matthews DR Use and abuse of HOMA modeling Diabetes Care 2004 27 1487 1495 10.2337/diacare.27.6.1487 15161807

[B37] Lee Y Hirose H Ohneda M Johnson JH McGarry JD Beta-cell lipotoxicity in the pathogenesis of non-insulin-dependent diabetes mellitus of obese rats: impairment in adipocyte-beta-cell relationships Proc Natl Acad Sci USA 1994 91 10878 10882 10.1073/pnas.91.23.10878 7971976 PMC45129

[B38] Pick A Clark J Kubstrup C Levisetti M Pugh W Role of apoptosis in failure of beta-cell mass compensation for insulin resistance and beta-cell defects in the male Zucker diabetic fatty rat Diabetes 1998 47 358 364 10.2337/diabetes.47.3.358 9519740

[B39] Smith SA Lister CA Toseland CD Buckingham RE Rosiglitazone prevents the onset of hyperglycaemia and proteinuria in the Zucker diabetic fatty rat Diabetes Obes Metab 2000 2 363 372 10.1046/j.1463-1326.2000.00099.x 11225966

[B40] Sudre B Broqua P White RB Ashworth D Evans DM Haigh R Junien JL Aubert ML Chronic inhibition of circulating dipeptidyl peptidase IV by FE 999011 delays the occurrence of diabetes in male zucker diabetic fatty rats Diabetes 2002 51 1461 1469 10.2337/diabetes.51.5.1461 11978643

