# Supplementary material for: Treatment with CNX-011-67, a novel GPR40 agonist, delays onset and progression of diabetes and improves beta cell preservation and function in male ZDF rats
Source: BMC Pharmacol Toxicol. 2013 May 21;14:28. doi: 10.1186/2050-6511-14-28 (PMC3668190; doi:10.1186/2050-6511-14-28)
Supplement: Additional file 1: Figure S1 — CNX-011-67 is highly specific to GPR40. [file 2050-6511-14-28-S1.doc]

**Supplementary Fig. 1. CNX-011-67 is highly specific to GPR40- studies with GPR40 specific shRNA in hGPR40-CHO-K cells and NIT-1 cells**


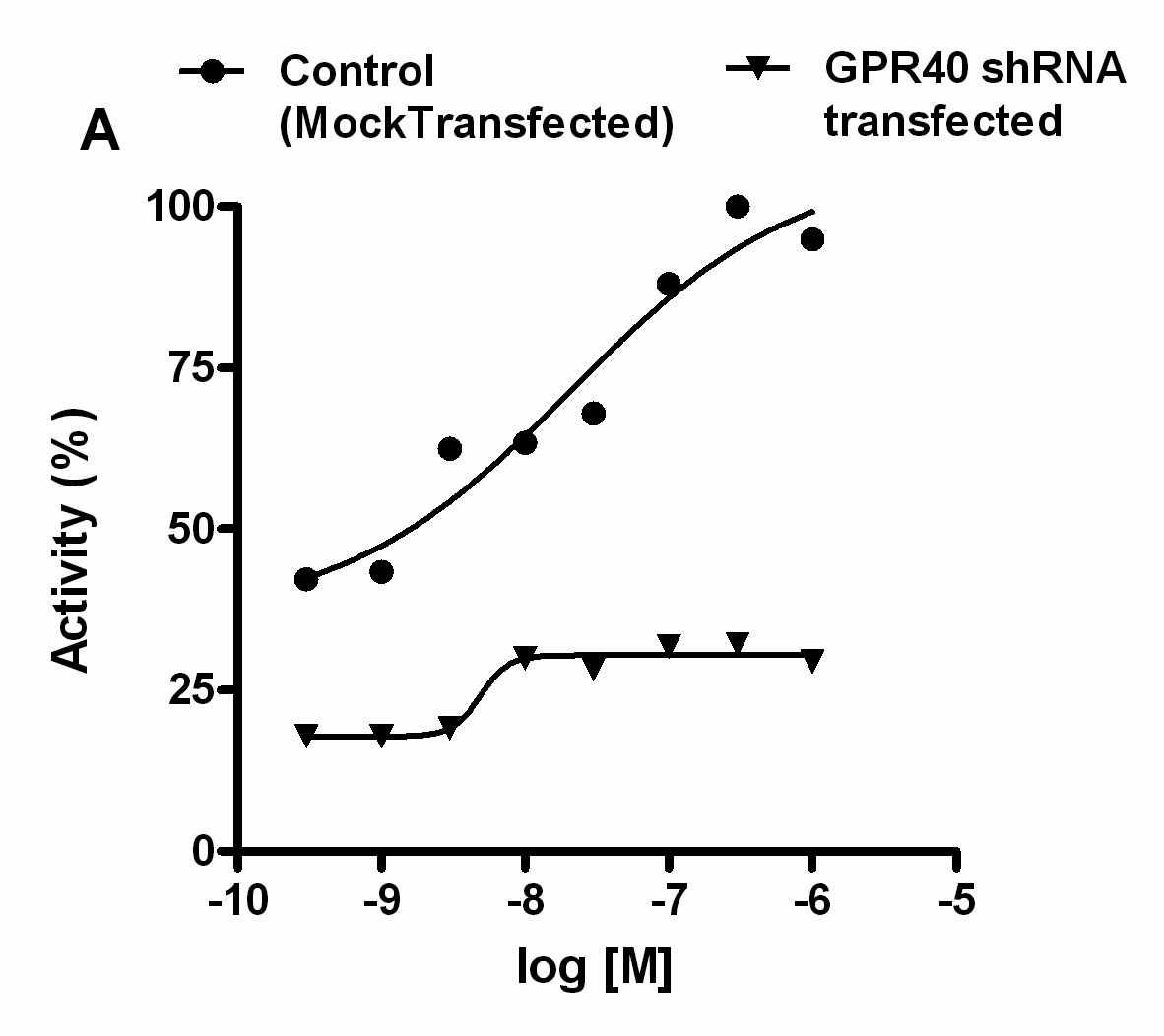


**
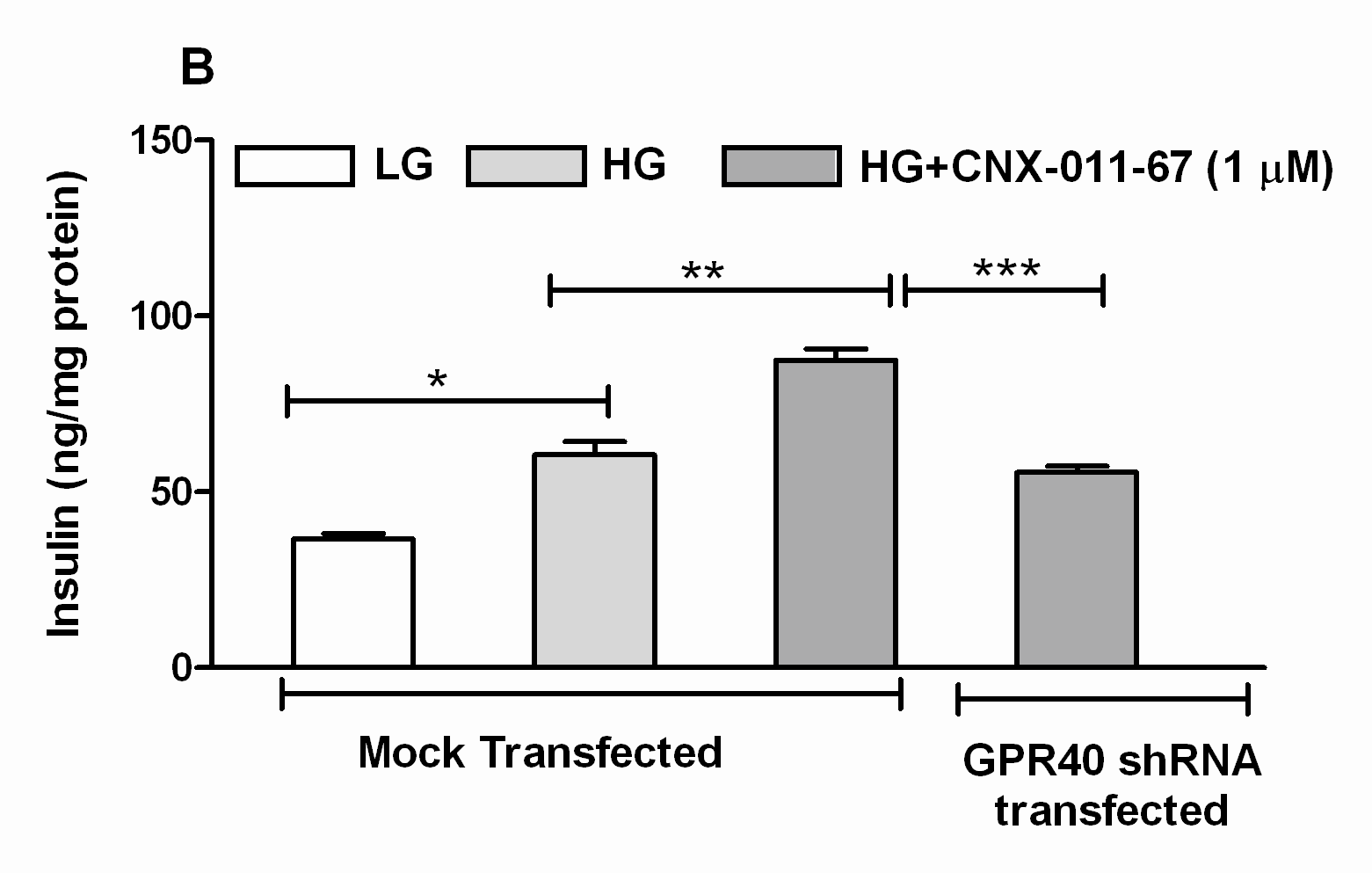
**

**Supplementary Fig. 1: (A)** The Effect of CNX-011-67 is mediated through GPR40. Recombinant hGPR40-CHO-K cells were transfected with scrambled shRNA and human GPR40-specific shRNA (Origene, USA), incubated for 24 h and then re-seeded. Calcium Flux assay was performed using Fluo-4 No-wash dye (Invitrogen). Cells were estimated for GPR40 knockdown using RT-PCR and an average inhibition of 60% was observed. For Calcium flux, following a basal measurement, cells were treated with CNX-011-67 at different concentrations followed by measurement of Ca^2+^ levels for 4 min at 6-s intervals. The resulting flux was converted to percentage activity (*n* = 3). Cells treated with GPR40 shRNA showed severe attenuation of Calcium flux in both magnitude and intensity compared to control cells. **(B)** NIT-1 cells were transfected with mGPR40-specific shRNA (Origene, USA) as mentioned above and used for acute GSIS assays. The control cells (mock transfected) showed elevation in Insulin secretion with CNX-011-67 treatment, the effect being completely abolished in mGPR40-specific shRNA transfected cells, indicating the role of GPR40 in mediating the effect of CNX-011-67. Secreted insulin is presented as ng of insulin/mg protein. Values represent mean ± SEM (*n* = 4). ***p*<0.01, ****p*<0.001 vs. corresponding control.

**Supplementary materials and methods:**

1. **shRNA studies in NIT-1 cells for GSIS**

NIT-1 cells were cultured in Ham’s-F12 media supplemented with 15% FBS. Semi-confluent cells were subcultured and plated at 0.6x10^6^ cells/well of a 6-well plate and grown to confluency of 70% for 72 h followed by a second media change and incubation for 24 h at 37°C/5% CO_2_. Cells were transfected with 1µg of shRNA plasmid contructs as per the manufacturer’s (Origene) protocol. The cells were incubated for 4 h, follwoed by a media change and further incubation for 24 h at at 37°C/5% CO_2_. A mock-transfected control was maintained for all experiments. Cells thus transfected were trypsinised, counted and reseeded at 4x10^4^ cells/well of a 96-well plate in Hams-F12-15% FBS media and incubated at 37°C/5% CO_2_ for 48h to reach 70% confluency. For Insulin ELISA, the medium was removed and cells were washed with 200μl KRBH (glucose-free) once and preincubated in Glucose-free-KRBH buffer for 30 minutes at 37°C/5% CO_2_. The KRBH medium was discarded and cells were again incubated in 100 μl of KRBH buffer with Glucose (2.5mM glucose and 16.7mM glucose along with drug) for 2h at 37°C/5% CO_2_. After 2h, the supernatant was removed from the cells and stored at -70°C for assaying insulin using a commercial ELISA (Mercodia). After removal of the supernatant, cells were washed with PBS solution, lysed with 50 μl of 0.1N Sodium hydroxide, and subjected to one freeze-thaw cycle for 1 h at -70°C. Protein estimation was performed using the Bradford method. Insulin secretion was represented as ng Insulin/mg protein.

1. **shRNA studies in recombinant hGPR40-CHO-K cells**

hGPR40-CHO-K cells were procured from Cytobox^TM^ (Germany) and used between passage numbers 3-10. Cells were cultured in Ham’s-F12 media supplemented with 10% FBS. Semi-confluent cells were subcultured and plated at 0.5x10^5^ cells/well of a 6-well plate and grown to confluency of 90% for 24h at 37°C/5% CO_2_. Cells were transfected with 2 µg of shRNA plasmid contructs as per the manufacturer’s (Origene) protocol. The cells were incubated for 4 h, followed by a media change and further incubation for 24 h at at 37°C/5% CO_2_. A mock-transfected control was maintained for all experiments. Cells thus transfected were trypsinised, counted and reseeded at 4x10^4^ cells/well of a 96-W plate in Hams-F12-10% FBS media containg 4 μg/ml G418 and incubated at 37°C/5% CO_2_ for 24h to reach 100% confluency. Fluo-4 No-Wash kit (Invitrogen) was used for the loading of CHO-K cells as per manufacturer’s instructions. Confluent (hGPR40-CHO-K and knockdown) cells were loaded with 90 µl of Fluo-4 dye and incubated at 37°C/5% CO_2_ for 1 h in dark. The cells were removed and transferred into BioTek Synergy reader previously calibrated for a fluorescent read at Ex.485/Em.525 nm for 5 minutes. A basal read was taken for 1 min. Following this, the drug was added as a 10X stock. After a stir cycle of 10s, readings were taken in the kinetic mode every 6 s for 5 min, and raw values were expressed as arbitrary fluorescence units. Values were expressed in terms of percentage activity and Cells were confirmed for GPR40 knockdown by gene expression analyses.

**Usage of Fluo-4 AM for cytosloic Calcium flux and Calcium Flux representation as AFU instead of Calcium Calibration.**

Due to the advantages of enhanced loading, reduced leakage and improved quantum yield Fluo-3 has been used in our experiments as a qualitative indicator of intracellular Calcium Flux changes. In-vitro Calcium level calibrations have been performed as per the equation

[Ca^2+^]i = K_d_[(F -F_min_)/(F_max_ - F)],

where Kd is the dissociation constant (400 nM for fluo-3), F is the fluorescence at cellular Ca^2+^ levels (after background fluorescence correction), F_min_ is the fluorescence intensity of the indicator in the absence of Ca^2+^and is obtained by adding a solution of 10 mM EGTA for 3 min, and F_max_ is the fluorescence of the Ca^2+^-saturated indicator and is obtained by adding a solution of 1 μM ionomycin for 3 min.

**Additional references:**

[Liu, Y.Q](http://www.ncbi.nlm.nih.gov/pubmed?term=Liu YQ%5BAuthor%5D&cauthor=true&cauthor_uid=12147706)., [Jetton, T.L](http://www.ncbi.nlm.nih.gov/pubmed?term=Jetton TL%5BAuthor%5D&cauthor=true&cauthor_uid=12147706)., [Leahy, J.L](http://www.ncbi.nlm.nih.gov/pubmed?term=Leahy JL%5BAuthor%5D&cauthor=true&cauthor_uid=12147706), 2002. beta-Cell adaptation to insulin resistance. Increased pyruvate carboxylase and malate-pyruvate shuttle activity in islets of nondiabetic Zucker fatty rats**.** [*J Biol Chem.*](http://www.ncbi.nlm.nih.gov/pubmed/12147706) 18, 39163-8
